# The influence of teacher-student relationship on primary school students' moral consensus: a moderated mediation model

**DOI:** 10.3389/fnbeh.2026.1723489

**Published:** 2026-02-04

**Authors:** Yao Wang, Jie Chen, Huiqing Chang, Guangming Li

**Affiliations:** 1 Institute of Education Sciences, Qiannan Normal University for Nationalities, Duyun, China; 2 School of Education, South China Normal University, Guangzhou, China; 3 School of Psychology, Center for Studies of Psychological Application, Guangdong Key Laboratory of Mental Health and Cognitive Science, South China Normal University, Guangzhou, China; 4 Key Laboratory of Brain, Cognition and Education Sciences (South China Normal University), Ministry of Education, Guangzhou, China

**Keywords:** grateful disposition, mindfulness, moral consensus, primary school students, social and emotional skills, teacher-student relationship

## Abstract

**Objective:**

This study aimed to examine the impact of the teacher-student relationship on moral consensus among primary school students, and to investigate the roles of mindfulness, grateful disposition, and social and emotional skills in this relationship.

**Methods:**

Using a cluster sampling method, 2,800 students from grades 3 to 5 across three schools in Guizhou Province completed collective questionnaires offline. Data were analyzed using SPSS 27.0 and the SPSS macro PROCESS to test the hypothesized model. The significance of all regression coefficients was tested using the Bootstrap method.

**Results:**

The teacher-student relationship significantly and positively predicted moral consensus (c = 0.48, *SE* = 0.02, *p* < 0.001). Social and emotional skills and grateful disposition partially mediated the relationship between the teacher-student relationship and moral consensus (a_1_b_1_ = 0.12, *Boot SE* = 0.01; a_2_b_2_ = 0.08, *Boot SE* = 0.01). Mindfulness significantly moderated the first part of the mediation pathway involving the teacher-student relationship and social and emotional skills. As the level of mindfulness increased, the positive predictive effect of the teacher-student relationship on social and emotional skills strengthened (*B*_simple_ at high mindfulness = 0.50, *t* = 23.74, *p* < 0.001, increasing from *B*_simple_ = 0.41 at low mindfulness). However, the moderating effect of mindfulness was not significant when grateful disposition served as the mediator.

**Conclusions:**

These findings enhance the understanding of the mechanisms through which the teacher-student relationship influences moral consensus and provide practical implications for fostering moral consensus among primary school students.

## 1 Introduction

In the context of global information flows and evolving social values, children are exposed to risks of moral uncertainty that may impede their character development, while the moral crises witnessed in recent decades have further underscored the urgency of moral education (Hafizi, 2023). However, with the advent of the new media era, the extensive use of media tools such as mobile phones has had a significant impact on the formation of children's moral consensus. New media has altered the traditional environment for children's moral education, leading to a certain degree of value generalization and moral anomie (Xie et al., 2018). Therefore, understanding the current state and developmental trends of children's moral consensus in the context of new media, and exploring pathways to promote its formation based on their actual growth environment, is particularly important.

Moral consensus refers to the shared recognition and agreement among members of a society regarding moral norms and qualities, which gradually develops within a specific social context (Xie et al., 2018). Children's moral consensus denotes the degree of common understanding and acceptance they hold toward moral norms and values—that is, from the perspective of children, it explores the shared baseline morality and moral beliefs among them (Bayertz, 1994; Xie et al., 2018). Moral consensus plays a crucial role in helping children understand and accept differences, as well as guiding them in confronting complex moral challenges (Hafizi, 2023). Jürgen Habermas's theory of moral consensus primarily focuses on how normative consensus, which should be followed for value judgments in communicative practice, is formed and applied (Habermas, 1983), emphasizing the process of reaching consensus through rational dialogue and debate (Oh, 2024). The theory of moral consensus posits that effective communication is based on equal and open dialogue among participants; therefore, the formation of children's moral consensus is also achieved through public rational dialogue, in which discussions with significant others play a particularly important role (Verovšek and Burdman, 2024).

Meanwhile, significant Others Theory posits that among various social environmental factors, an individual's interactive relationships with significant figures play a crucial role in fulfilling basic psychological needs (Xu et al., 2025). Bronfenbrenner and Morris introduced the concept of “significant others” in their research on roles and interpersonal interactions within the microsystem to describe important individuals with whom children maintain regular interactions over a considerable period, primarily including parents, teachers, and friends (Bronfenbrenner and Morris, 2007). Research indicates that both the teacher-student relationship and the quality of children's friendships can positively predict children's moral sensitivity (Lu and Fu, 2021),higher moral sensitivity in children is associated with a higher level of moral identity (Xiang et al., 2022). The teacher-student relationship is a fundamental interpersonal connection formed through the interaction between teachers and students, serving as a fundamental relationship that influences children's behavior, beliefs, and patterns of feeling (Aldhafri and Alhadabi, 2019). Pianta categorized teacher-student relationships into three dimensions: closeness, conflict, and dependency. A close teacher-student relationship reflects an open, warm, and secure connection in which children perceive their teacher as a source of support. In contrast, a conflicted teacher-student relationship is characterized by negative, discordant, unpredictable, and unpleasant interactions between the teacher and the student (Pianta and Steinberg, 1992). Positive teacher-student relationship is a key factor in promoting student development and reducing problematic behaviors. It facilitates the development of students' moral character, the improvement of their academic performance, the formation of positive personality traits, and the enhancement of social adaptability (Zou et al., 2007).

Furthermore, a significant positive correlation exists between the teacher-student relationship and the social and emotional skills of primary school students (Deng et al., 2024). A close teacher-student relationship can promote the development of students' social and emotional skills and prosocial behaviors such as grateful disposition (Wu and Zhang, 2022). Social and emotional competence (SEC) refers to the ability to understand, manage, and express the social and emotional aspects of one's life. Individuals with well-developed SEC can effectively recognize and control their emotions, develop care and concern for others, successfully manage life tasks, adapt to the complex demands of growth and development, and effectively handle various difficulties and challenges they encounter (Weissberg et al., 2015). Substantial empirical research demonstrates that students' social and emotional skills have a significant positive impact on the development of their prosocial behaviors (Zhou and Deng, 2025), and prosocial behaviors are significantly correlated with moral self-perception (Zhao et al., 2024). A close connection exists between social and emotional skills and morality. Well-developed social and emotional skills allow individuals to better understand others' perspectives and emotions, thereby enabling them to respect others, maintain rationality in the face of difficulties and setbacks, and better adhere to moral principles.

Further research indicates that gratitude also influences prosocial behavior and moral decision-making among primary and secondary school students (Yao, 2024; Huang, 2023). Compared with individuals with a lower disposition toward gratitude, those with a higher tendency demonstrate stronger prosocial inclinations, more nuanced moral judgment, and a more robust moral identity (Lian, 2024). Gratitude is defined as a generalized psychological tendency to recognize and acknowledge the help or benefits received from others and to feel motivated to reciprocate (McCullough et al., 2002). Research by McCullough and colleagues suggests that gratitude, as a moral emotion, shares similarities with other moral emotions such as empathy and guilt, and functions as an affective state linked to cognition and behavior.

A study conducted on adults aged 18 and above found that mindfulness significantly predicts grateful disposition (Voci et al., 2019). This is consistent with other findings showing that individuals with higher levels of mindfulness also report greater degrees of grateful disposition (Li, 2020). Mindfulness is a psychological state of concentrating attention on present-moment experiences, consciously and non-judgmentally aware of one's immediate state and feelings (Zhang, 2024). Substantial research demonstrates that mindfulness training can promote adolescents' mental health, improve their emotional state, and enhance emotion regulation abilities (Liu et al., 2019). For instance, the CARE program in the United States focuses on this aspect, utilizing mindfulness training to help teachers alleviate stress and enhance their social and emotional skills (Song and Chang, 2022). Over the past decade, the relationship between mindfulness and morality has garnered widespread attention. Studies have confirmed that mindfulness plays a significant role in moral behavior (Guo et al., 2022), and individuals with high trait mindfulness are more likely to exhibit high levels of moral competence in moral dilemmas and moral behaviors (Chen and Wang, 2017).

**Figure 1:**
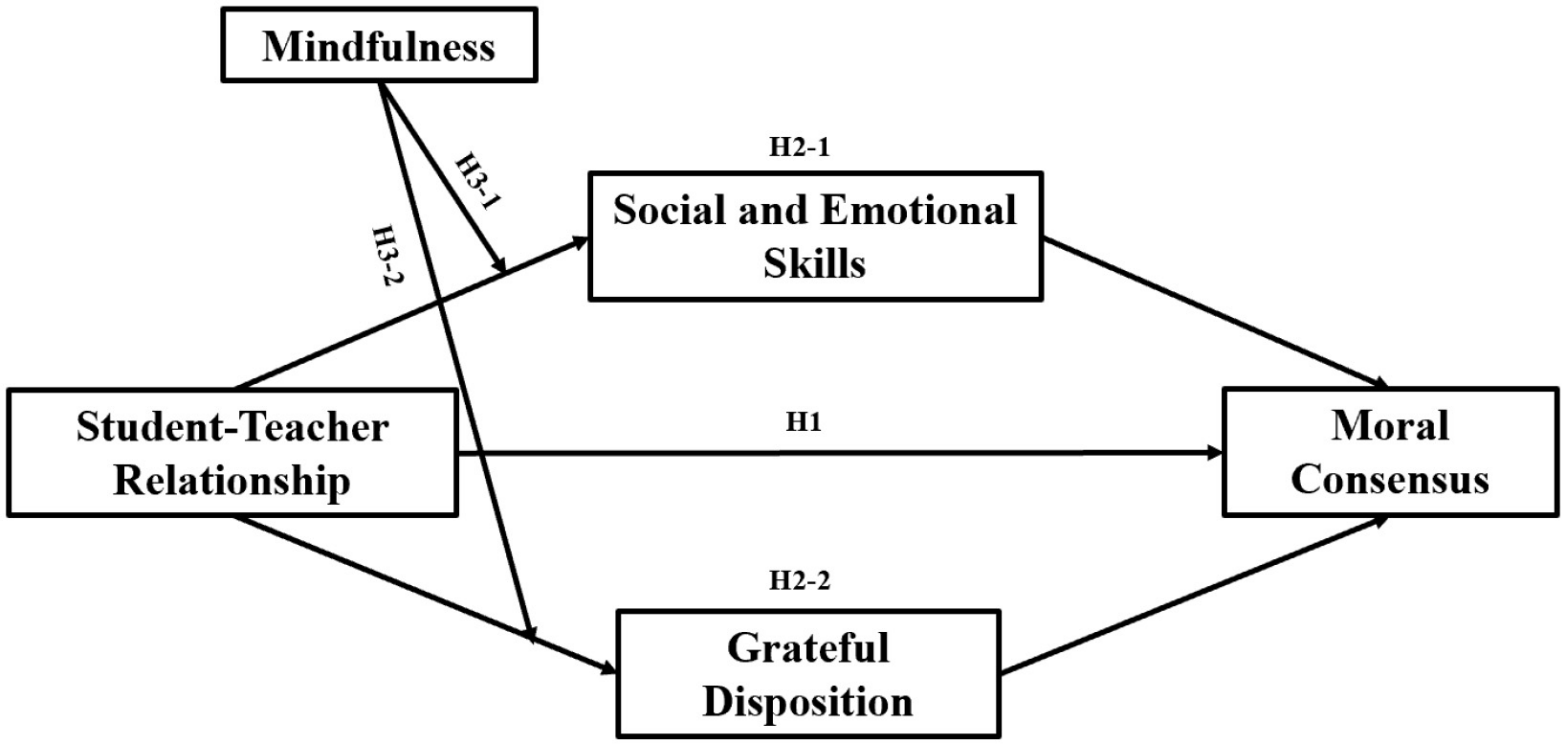
Diagram of a moderated mediation model.

In conclusion, the link between the recently developed Moral Consensus Scale and teacher-student relationships remains unexplored. Since teachers are key influencers in student development, their role in shaping students' moral consensus is fundamentally important. According to Lawrence Kohlberg's theory of moral development, during the conventional stage (ages 9-15), children's moral values are oriented toward interpersonal harmony, emphasizing rules and obligations, making it a critical period for fostering moral consensus (Wang, 2015). Therefore, this study aims to construct a moderated mediation model to explore the relationship between the teacher-student relationship and moral consensus among primary school students, as well as the roles of social and emotional skills, grateful disposition, and mindfulness within this relationship (Figure 1). The following hypotheses are proposed:

**H1**: The quality of the teacher-student relationship positively predicts the level of moral consensus among primary school students.
**H2-1**: Social and emotional skills mediate the relationship between the teacher-student relationship and moral consensus.
**H2-2:** Grateful disposition mediates the relationship between the teacher-student relationship and moral consensus.
**H3-1**: Mindfulness moderates the first part of the mediation path (i.e., the path from the teacher-student relationship to social and emotional skills).
**H3-2**: Mindfulness moderates the first part of the mediation path (i.e., the path from the teacher-student relationship to grateful disposition).

## 2 Methods

### 2.1 Participants and procedure

This study utilized a cluster sampling method. Questionnaires were administered offline to students from grades 3 to 5 across three schools in Guizhou Province. A total of 2,800 questionnaires were distributed. After excluding blank and incomplete responses, 2,741 valid questionnaires were obtained, yielding a valid response rate of 97.89%. The sample consisted of 1,490 boys (54.36%) and 1,251 girls (45.64%), with a mean age of 10.12 years (*SD* = 0.84).

### 2.2 Measures

#### 2.2.1 Teacher-student relationship

The teacher-student relationship was assessed using the Teacher-Student Relationship Scale (TSRS) developed by Pianta (2001) and revised by Zou et al. (2007). This scale has been validated for use with elementary school students in grades 3–5, a population typically aged 8 to 11 years (Ai et al., 2024). The scale consists of 23 items measured on a 5-point Likert scale, ranging from 1 (“completely untrue”) to 5 (“completely true”). A higher total score indicates a higher quality of the teacher-student relationship. The scale encompasses four dimensions: closeness, support, satisfaction, and conflict. It has demonstrated good reliability and validity in previous studies. In the present study, the Cronbach's α coefficient was 0.92.

#### 2.2.2 Moral consensus

Moral consensus was measured using the Moral Consensus Scale developed by Xie et al. (2018), which was applicable to individuals aged 7 to 17. This 17-item scale comprises three dimensions: moral cognitive consensus, moral behavioral consensus, and moral emotional consensus. Responses were recorded on a 5-point Likert scale from 1 (“strongly disagree”) to 5 (“strongly agree”), with higher scores indicating a higher level of moral consensus among children. The scale has shown good psychometric properties. In this study, the Cronbach's α coefficient was 0.90.

#### 2.2.3 Social and emotional skills

Social and emotional skills were measured using the 10-year-old version of the Social and Emotional Skills Assessment Scale developed by the Organization for Economic Co-operation and Development (Organisation for Economic Cooperation and Developmental, 2021). This scale has been verified to be applicable in the measurement of 8 year old children (Kogler et al., 2025). The scale comprises 31 items divided into five dimensions: collaboration skills (e.g., “I like to help others”), social engagement skills (e.g., “I have many friends”), emotional regulation skills (e.g., “I try to see the good side when something happens”), task performance skills (e.g., “I finish what I start”), and open-mindedness (e.g., “I enjoy learning new things”). Each item is rated on a 5-point Likert scale, where 1 represents “strongly disagree” and 5 represents “strongly agree.” Higher scores indicate better social and emotional skills (Wang, 2024). In the present study, the scale demonstrated good internal consistency, with a Cronbach's α coefficient of 0.88.

#### 2.2.4 Grateful disposition

The Grateful Disposition Questionnaire (GQ-6), developed by McCullough et al. (2002), was used to assess individual differences in the tendency to experience grateful disposition in daily life. The GQ-6 scale has been applied to children aged 8–10 years with good measurement outcomes (Chen et al., 2025; Wei et al., 2016; Liang et al., 2022). The 6-item instrument employs a 7-point Likert scale, with higher total scores indicating a greater propensity for grateful disposition. In this study, the Cronbach's α coefficient for the GQ-6 was 0.81.

#### 2.2.5 Mindfulness

Mindfulness was assessed using the Child and Adolescent Mindfulness Measure (CAMM), originally developed by Greco et al. (2011). And subsequently revised for the Chinese context by Liu et al. (2019). This scale has been validated in children aged 7-12 (García-Rubio et al., 2019). The 10-item scale consists of two dimensions: awareness and non-judgment, and acceptance. Items are rated on a 5-point frequency scale ranging from 1 (“never”) to 5 (“always”). Higher total scores reflect a greater level of mindful awareness. For the current sample, the measure showed acceptable reliability, with a Cronbach's α coefficient of 0.81.

### 2.3 Data analysis

This study used SPSS 27.0 and the SPSS macro PROCESS developed by Hayes (2013) to test the hypothesized model. The significance testing for all regression coefficients was conducted using the Bootstrap method.

First, during the data processing stage, necessary data cleaning and descriptive statistics were performed on all scale data. To test for common method bias in the questionnaires, following Podsakoff's et al. (2003) recommendation, Harman's single-factor test was employed. The fundamental rationale of this method is to include all measurement items simultaneously into an unrotated factor analysis; it is then determined whether the variance explained by the first factor exceeds 40%. The correlation coefficient between teacher-student relationship and moral consensus can be used to test Hypothesis H1.

Subsequently, we proceeded to examine the mechanism through which the independent variable (X) influences the dependent variable (Y) via the mediating variable (M) (i.e., Hypotheses H2-1 and H2-2). According to research by Hayes (2013) and Wen and Ye, 2014b, Model 4 of the PROCESS plugin for SPSS can be used to test simple mediating effects. This plugin can utilize the bootstrapping method to estimate the path coefficients of the model, the mediating effect, the moderated mediating effect, and their confidence intervals. Based on this, we determine whether the mediation is full mediation (direct effect is not significant) or partial mediation (direct effect remains significant) by testing the significance of the direct effect.

If the mediating effect is significant, we then begin to test how the moderating variable (W) influences the mediating effect of the mediator (i.e., Hypotheses H3-1 and H3-2). According to research by Hayes (2013) and Wen and Ye (2014a), Model 7 of the PROCESS plugin for SPSS can be used to test a moderated mediation model where the moderator variable affects the first stage of the mediation path. If the coefficient of the interaction term between the independent variable and the moderating variable is significant, it indicates a significant moderating effect. To visually present the moderated mediating effect, we will provide a moderation effect test table and plot the interaction effect of the moderating variable. Furthermore, based on research by Liu (2001), we use the Variance Inflation Factor (VIF) indicator to assess whether serious multicollinearity exists in the model.

## 3 Results

### 3.1 Common method bias test

The Harman's single-factor test was used to examine potential common method bias. The results indicated that there were 17 factors with eigenvalues greater than 1. The first factor explained 18.76% of the total variance, which is below the critical threshold of 40%. Therefore, no significant common method bias was detected in this study.

### 3.2 . Descriptive statistics and correlation analysis

#### 3.2.1 Correlation analysis of variables

Table 1 presents the means, standard deviations, and correlation matrix for the study variables. The results revealed that grateful disposition, teacher-student relationship, moral consensus, mindfulness, and social and emotional skills were all significantly and positively correlated with each other. The correlation analysis also indicated that sex and grade were significantly correlated with the main study variables. Consequently, both sex and grade were treated as control variables in the subsequent data analyses.

**Table 1:** Descriptive statistics and correlation matrix for all variables.

**Factors**	***M***	***SD***	**1**	**2**	**3**	**4**	**5**	**6**
1. Sex	1.46	0.50						
2. Grade	2.02	0.82	0.01					
3. Grateful Disposition	5.20	1.14	−0.01	−0.01				
4. Teacher-Student Relationship	3.55	0.69	0.08^***^	−0.08^***^	0.46^***^			
5. Moral Consensus	4.14	0.653	0.08^***^	−0.13^***^	0.36^***^	0.49^***^		
6. Mindfulness	3.52	0.76	0.04	−0.03	0.21^***^	0.34^***^	0.34^***^	
7. Social and Emotional Skills	3.39	0.53	−0.03	−0.15^***^	0.37^***^	0.53^***^	0.43^***^	0.37^***^

Sex (1 = male, 2 = female) and grade (1 = third grade, 2 = fourth grade, 3 = fifth grade) were treated as categorical variables. N = 2,741.

p^***^ < 0.001. The same applies to the following tables.

#### 3.2.2 The role of social and emotional skills: testing the moderated mediation model

In accordance with the perspectives of Hayes (2013) and Wen and Ye (2014b), the simple mediation effect of social and emotional skills was tested using Model 4 from the SPSS macro PROCESS, and the moderating effect of mindfulness within this relationship was tested using Model 7.

First, Model 4 was used to examine the mediating effect of social and emotional skills in the relationship between the teacher-student relationship and moral consensus. The results indicated that, after controlling for sex and grade, the teacher-student relationship significantly predicted moral consensus (c = 0.48, *SE* = 0.02, *p* < 0.001). The teacher-student relationship also significantly predicted social and emotional skills (a_1_ = 0.53, *SE* = 0.02, *p* < 0.001). When both the teacher-student relationship and social and emotional skills were included to predict moral consensus, the teacher-student relationship remained a significant predictor of moral consensus (${\text{c}}_{1}^{\prime }$ = 0.35, *SE* = 0.02, *p* < 0.001), and social and emotional skills significantly predicted moral consensus (b_1_ = 0.23, *SE* = 0.02, *p* < 0.001). The test using the bias-corrected percentile Bootstrap method showed that the indirect effect of the teacher-student relationship on moral consensus through social and emotional skills was significant (a_1_b_1_ = 0.12, *Boot SE* = 0.01, 95% CI [0.10, 0.15]). Therefore, social and emotional skills played a partial mediating role in the relationship between the teacher-student relationship and moral consensus, thus supporting Hypothesis H2-1.

In the second step, Model 7 of the PROCESS macro was used to test the moderating effect of mindfulness on the mediation pathway involving social and emotional skills. Equation 1 estimated the total effect of the teacher-student relationship on moral consensus; Equation 2 estimated the predictive effect of social and emotional skills on moral consensus; Equation 3 estimated the moderating effect of mindfulness on the relationship between the teacher-student relationship and social and emotional skills. All predictor variables were standardized in each equation. A moderated mediation effect is considered to exist if the model estimation meets the following three conditions: (a) in Equation 1, the total effect of the teacher-student relationship on moral consensus is significant; (b) in Equation 2, the predictive effect of social and emotional skills on moral consensus is significant; and (c) in Equation 3, the main effect of the teacher-student relationship on social and emotional skills is significant, and the interaction effect between the teacher-student relationship and mindfulness is significant. In this study, the variance inflation factor (VIF) for all predictor variables did not exceed 5, indicating no serious multicollinearity problems.

As shown in Table 2, in Equation 1, the total effect of the teacher-student relationship on moral consensus was significant, meeting condition (a); Equation 2 was significant, indicating the predictive effect of social and emotional skills on moral consensus was significant, meeting condition (b); Equation 3 was significant, showing that the teacher-student relationship positively predicted social and emotional skills, and the interaction term between the teacher-student relationship and mindfulness was significant, meeting condition (c). Therefore, it can be concluded that mindfulness moderates the mediating effect of social and emotional skills, thus supporting Hypothesis H3-1.

**Table 2:** Test of the moderating effect of mindfulness on the mediating role of social and emotional skills.

**Independent variables**	**Equation1**			**Equation2**			**Equation3**		
	**(Dependent variable: moral consensus)**			**(Dependent variable: moral consensus)**			**(Dependent variable: social and emotional skills)**		
	β	***SE***	***t***	β	***SE***	***t***	β	***SE***	***t***
Sex	0.09^**^	0.03	2.59	0.12^***^	0.03	3.66	−0.15^***^	0.03	−4.73
Grade	−0.12^***^	0.02	−5.84	−0.09^***^	0.02	−4.36	−0.13^***^	0.02	−7.00
Teacher-Student Relationship	0.49^***^	0.02	29.15	0.35^***^	0.02	18.32	0.45^***^	0.02	27.61
Social and Emotional Skills				0.23^***^	0.02	11.99			
Mindfulness							0.21^***^	0.02	12.89
Social and Emotional Skills ^*^ Mindfulness							0.04^**^	0.01	3.25
R^2^	0.25^***^			0.29^***^			0.35^***^		
F	300.70			273.22			288.96		

^*^p < 0.05, ^**^p < 0.01, ^***^p < 0.001.

**Figure 2:**
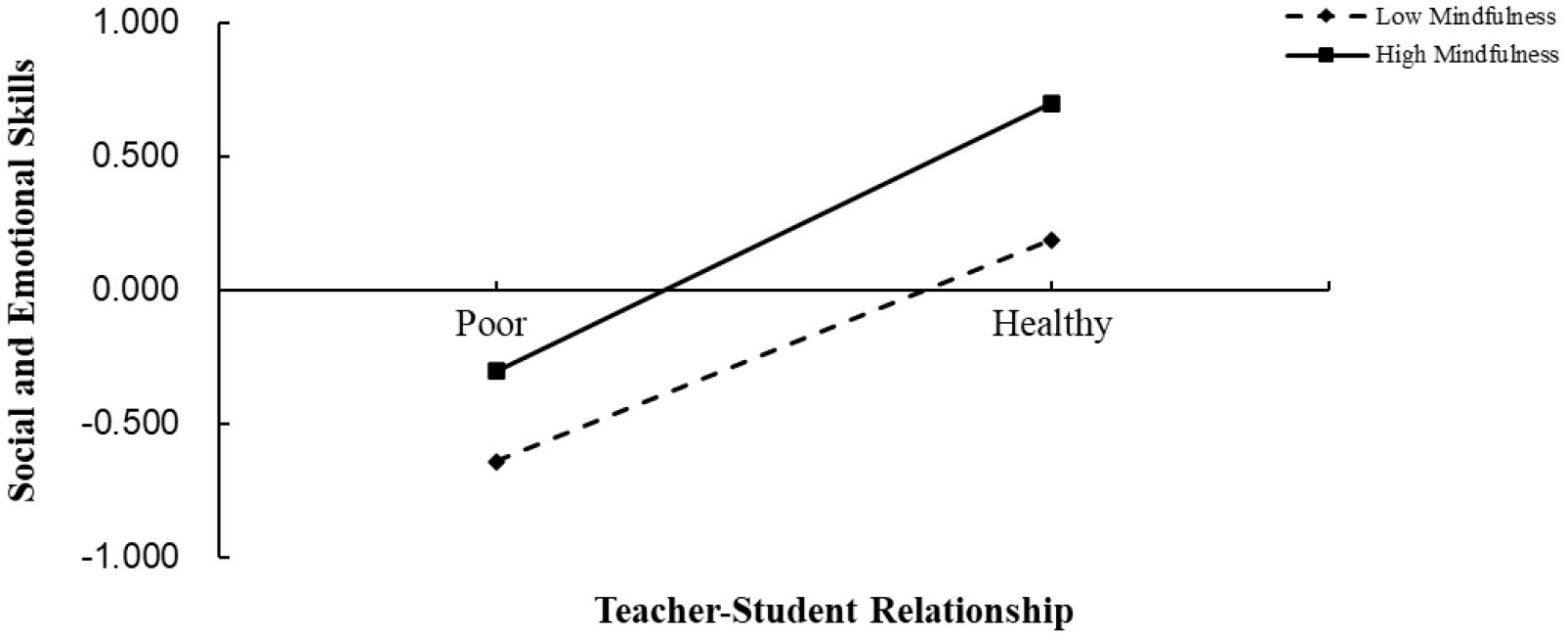
The moderating role of mindfulness in the relationship between teacher-student relationship and social-emotional skills.

To further clarify the nature of the interaction effect between the teacher-student relationship and mindfulness, a simple slope test was conducted by grouping participants based on mindfulness scores one standard deviation above and below the mean (high and low groups), and a simple effect analysis graph was plotted (see Figure 2). The results showed that for the low mindfulness group, the teacher-student relationship significantly and positively predicted social and emotional skills (*B*_simple_ = 0.41, *t* = 19.27, *p* < 0.001). For the high mindfulness group, the positive predictive effect of the teacher-student relationship on social and emotional skills was enhanced (*B*_simple_ = 0.50, *t* = 23.74, *p* < 0.001), increasing from *B*_simple_ = 0.41 to *B*_simple_ = 0.50.

#### 3.2.3 . The role of grateful disposition: testing the moderated mediation model

The same testing procedure used for social and emotional skills was applied. First, Model 4 of the SPSS macro PROCESS was employed to examine the mediating effect of grateful disposition in the relationship between the teacher-student relationship and moral consensus. The results indicated that, after controlling for sex and grade, the teacher-student relationship significantly predicted moral consensus (c = 0.48, *SE* = 0.02, *p* < 0.001). The teacher-student relationship also significantly predicted grateful disposition (a_2_ = 0.46, *SE* = 0.02, *p* < 0.001). When both the teacher-student relationship and grateful disposition were included in the model to predict moral consensus, the teacher-student relationship remained a significant predictor of moral consensus (${\text{c}}_{2}^{\prime }$ = 0.39, *SE* = 0.02, *p* < 0.001), and grateful disposition significantly predicted moral consensus (b_2_ = 0.18, *SE* = 0.02, *p* < 0.001). The test using the bias-corrected percentile Bootstrap method showed that the indirect effect of the teacher-student relationship on moral consensus through grateful disposition was significant (a_2_b_2_ = 0.08, *Boot SE* = 0.01, 95% CI [0.07, 0.10]). Therefore, grateful disposition played a partial mediating role in the relationship between the teacher-student relationship and moral consensus, thus supporting Hypothesis H2-2.

In the second step, Model 7 of the SPSS macro PROCESS was used to test the moderating effect of mindfulness on the mediation pathway involving grateful disposition. Equation 1 estimated the total effect of the teacher-student relationship on moral consensus; Equation 4 estimated the predictive effect of grateful disposition on moral consensus; Equation 5 estimated the moderating effect of mindfulness on the relationship between the teacher-student relationship and grateful disposition. All predictor variables were standardized in each equation. A moderated mediation effect is considered to exist if the model estimation meets the following three conditions: (a) in Equation 1, the total effect of the teacher-student relationship on moral consensus is significant; (b) in Equation 2, the predictive effect of grateful disposition on moral consensus is significant; (c) in Equation 3, the main effect of the teacher-student relationship on grateful disposition is significant, and the interaction effect between the teacher-student relationship and mindfulness is significant.

As shown in Table 3, in Equation 1, the total effect of the teacher-student relationship on moral consensus was significant, satisfying condition (a). Equation 4 was significant, indicating that the predictive effect of grateful disposition on moral consensus was significant, satisfying condition (b). Equation 5 was significant, showing that the teacher-student relationship positively predicted grateful disposition; however, the interaction term between the teacher-student relationship and mindfulness was not significant, did not satisfy condition (c). Since the sequential test was not passed, the product-of-coefficients interval test was further employed. The results showed that the 95% confidence interval for the index of the moderated mediation effect was [-0.01,−0.01]. As this confidence interval included zero, the interval test also failed. Therefore, it was concluded that the moderated mediation effect was not significant. Mindfulness neither moderated the predictive effect of the teacher-student relationship on grateful disposition nor moderated the mediating role of grateful disposition in the relationship between the teacher-student relationship and moral consensus. Thus, hypothesis H3-2 was rejected.

**Table 3:** Test of the moderating effect of mindfulness on the mediating role of gratitude.

**Independent variables**	**Equation1**			**Equation4**			**Equation5**		
	**(Dependent variable: moral consensus)**			**(Dependent variable: moral consensus)**			**(Dependent variable: mindfulness)**		
	β	***SE***	***t***	β	***SE***	***t***	β	***SE***	***t***
Sex	0.09^**^	0.03	2.59	0.10^**^	0.03	3.12	-0.09^**^	0.03	-2.65
Grade	-0.12^***^	0.02	-5.84	0.02	-6.24		0.03	0.02	1.64
Teacher-Student Relationship	0.49^***^	0.02	29.15	0.41^***^	0.02	22.02	0.44^***^	0.02	24.25
Grateful Disposition				0.18^***^	0.02	9.54			
Mindfulness							0.06^***^	0.02	3.47
Teacher-Student Relationship^*^Mindfulness							-0.01	0.02	-0.01
R^2^	0.25^***^			0.27^***^			0.21^***^		
F	300.70			258.36			148.74		

^*^p < 0.05, ^**^p < 0.01, ^***^p < 0.001.

#### 3.2.4 . Moderated mediation effects

**Figure 3:**
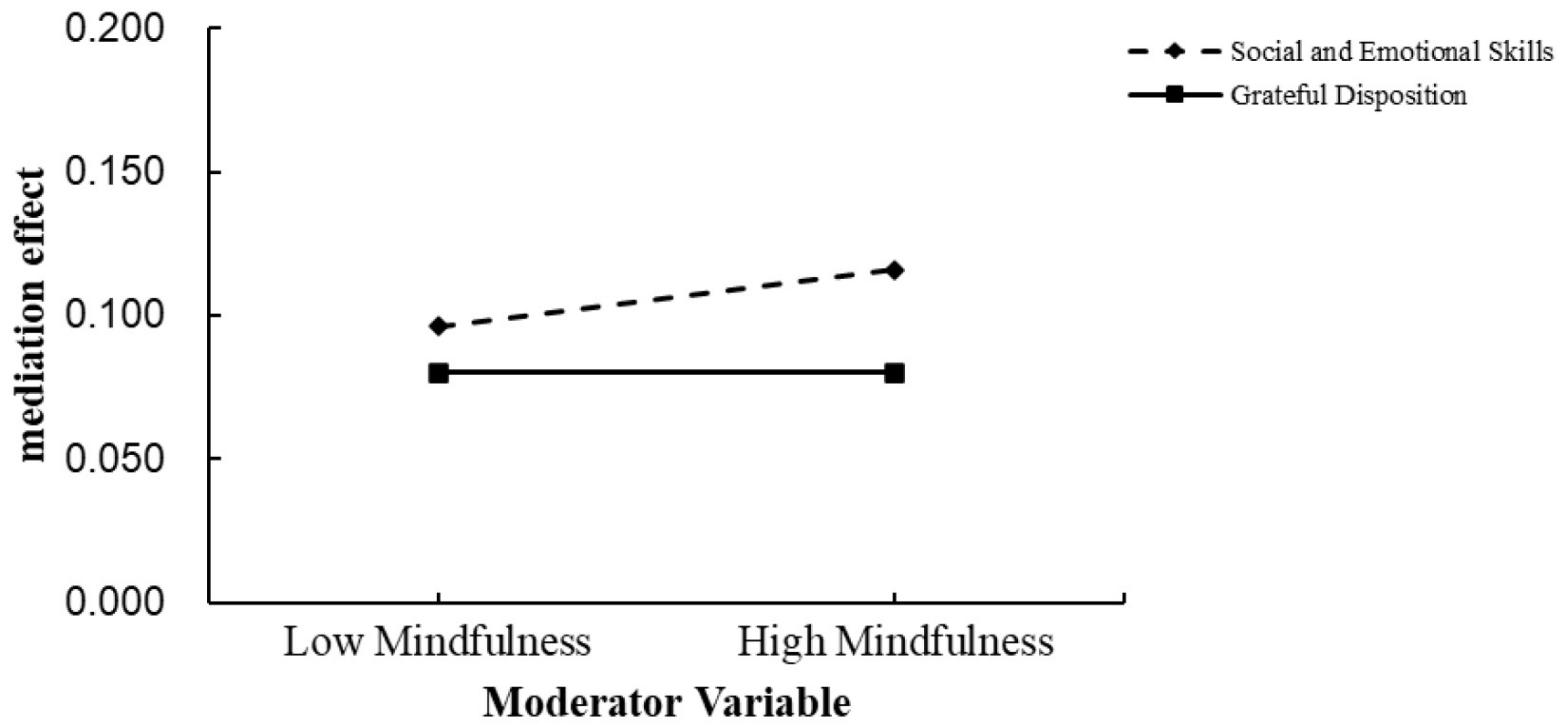
Figure of the moderating effect of mindfulness on mediation effect.

To more clearly illustrate the moderating effect of mindfulness on the mediation pathways, mindfulness was divided into high and low groups based on scores one standard deviation above and below the mean. A mediation effect analysis graph was created (see Figure 3). As shown in Figure 3, when social and emotional skills served as the mediator, the moderated mediation effect was significant (index = 0.01, 95% CI [0.00, 0.02]). However, when grateful disposition served as the mediator, the moderated mediation effect was not significant (index = 0.00, 95% CI [-0.01, 0.01]).

**Figure 4:**
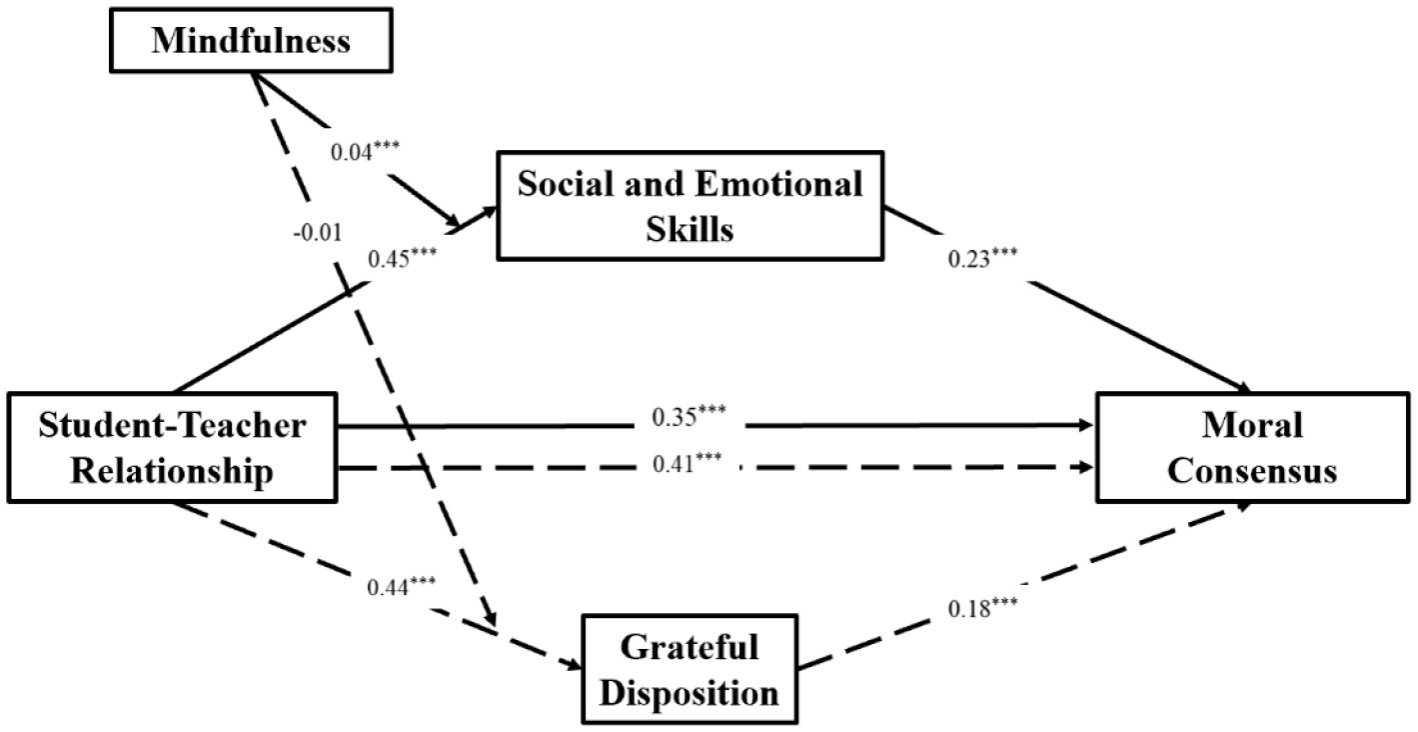
Path diagram.

Based on the analysis results of PROCESS Model 7, a path coefficient diagram (Figure 4) was created to provide a more intuitive representation of the relationships among the variables and the mediation and moderation effects.

## 4 Discussion

### 4.1 The influence of teacher-student relationship on moral consensus

The results of this study indicate that the teacher-student relationship significantly and positively predicted the moral consensus of primary school students. According to Kohlberg's theory of moral development, during the conventional stage (ages 9-15), children's moral values are oriented toward interpersonal harmony, emphasizing rules and obligations, making it a critical period for fostering moral consensus. Teachers are key influencers in student development, their role in shaping students' moral consensus is fundamentally important. As an important interpersonal relationship for children, the teacher-student relationship plays a key role in fulfilling basic psychological needs (Wang, 2015). Existing research has shown that the teacher-student relationship is an important predictor of children's moral sensitivity. A close and harmonious teacher-student relationship promotes the development of a higher level of moral sensitivity in children, while a poor teacher-student relationship may lead to problems in children's moral behavior (Lu and Fu, 2021).

Habermas's theory of moral consensus posits that the normative consensus which should be followed for value judgments is formed and applied in communicative practice (Habermas, 1983), emphasizing the process of reaching consensus through rational dialogue and debate (Oh, 2024).

Therefore, the formation of children's moral consensus is also achieved based on communicative practice and public rational dialogue (Verovšek and Burdman, 2024). Through accumulated social interactions over time, children learn to more subtly recognize and interpret moral situations and adopt appropriate actions.

### 4.2 The mediating roles of social and emotional skills and grateful disposition?

#### 4.2.1 The mediating role of social and emotional skills?

The results of this study indicate that social and emotional skills play a partial mediating role in the relationship between the teacher-student relationship and moral consensus. Hypothesis H2-1 is supported, and the findings are consistent with relevant theories and conclusions from prior research. First, this finding is consistent with results from global adolescent social and emotional skills assessment projects, which indicate a positive correlation between the teacher-student relationship and students‘ social and emotional skills (Organisation for Economic Cooperation and Developmental, 2021). A close teacher-student relationship can promote the development of primary school students' social and emotional skills (Deng et al., 2024) and their emotion regulation abilities (Pallini et al., 2019). According to the multiple attachment theory, a positive teacher-student relationship, with teachers acting as significant others and direct caregivers, can provide students with basic emotional security (Chen and Lin, 2021). Through close interactions with teachers, students enhance their emotional capacities in various areas such as communication, collaboration, empathy, and compassion (Zhang and Mao, 2020). Conversely, students with well-developed social and emotional skills can express their feelings and ideas more appropriately during interactions with teachers, thereby fostering a positive teacher-student relationship (Wang et al., 2021).

Second, well-developed social and emotional skills allow individuals to better understand others‘ perspectives and emotions, leading to greater respect for others, maintaining rationality in the face of difficulties and setbacks, and better adhering to moral principles (Zhou and Deng, 2025). Substantial empirical research demonstrates that students' social and emotional skills have a significant positive impact on the development of their prosocial behaviors, and prosocial behaviors are significantly correlated with moral self-perception (Zhao et al., 2024).

#### 4.2.2 The mediating role of grateful disposition

The results of this study indicate that grateful disposition played a partial mediating role in the relationship between the teacher-student relationship and moral consensus. The cognitive-emotion theory posits that grateful disposition is a positive emotion, and the generation of this emotion depends to some extent on the beneficiary's cognition and attribution of the benefactor's behavioral events (Emmons and McCullough, 2003). Research has shown that teachers‘ caring behaviors can effectively influence students' psychological capital, including grateful disposition (Zhao, 2023), and that teachers‘ emotional support is significantly positively correlated with students' grateful disposition (Qiang, 2024). The moral emotion theory, on the other hand, suggests that grateful disposition is a moral emotion that can promote the occurrence of moral behavior (McCullough et al., 2002). Grateful disposition influences the social goodwill of primary and secondary school students (Yao, 2024); compared to individuals with lower levels of grateful disposition, those with higher levels of grateful disposition demonstrate higher levels of social goodwill (Huang, 2023), more nuanced moral decision-making, and a stronger moral identity (Lian, 2024). In this study, grateful disposition played a partial mediating role between the teacher-student relationship and moral consensus, thus supporting Hypothesis H2-2, which is consistent with relevant theories and findings from existing research.

#### 4.2.3 . The moderating role of mindfulness?

The findings of this study indicate that the indirect effect of the teacher-student relationship on primary school students‘ moral consensus, through the mediation of social and emotional skills, is moderated by mindfulness. These results support Hypothesis H3-1 and are consistent with relevant theoretical frameworks and existing research. Research has shown that mindfulness training can promote adolescents' mental health, improve their emotional state, and enhance emotion regulation abilities (Newberg et al., 2001). Evidence suggests that during meditation, gray matter in the brain is activated, and this region is closely related to individual emotion regulation, perspective-taking, and perceptual attention (Hölzel et al., 2011). Simultaneously, mindfulness practice increases insula activity, a brain region significantly associated with processing the emotional experiences of others (Singer et al., 2009). According to the re-perceiving model proposed by Shapiro et al. (2006), when individuals face negative emotions, re-perceiving through mindfulness provides more flexible cognition and a more objective emotional experience. This new cognition can replace the emotional experience generated by pre-existing views or even biases in the mind (Brown et al., 2007).

However, in this study, the indirect effect of the teacher-student relationship on moral consensus through grateful disposition was not moderated by mindfulness. This might be because the children were too young, especially considering the very limited cognitive abilities of younger children. Young children may have difficulty truly understanding the abstract concepts of grateful disposition and mindfulness. Furthermore, the stress vulnerability hypothesis suggests that for individuals possessing certain positive psychological qualities (e.g., grateful disposition, honesty), their ability to resist risks can be greatly reduced when facing stressful life events that they cannot temporarily resolve (Fu et al., 2012). This suggests that higher levels of perceived stress may be associated with poorer adaptability in individuals possessing these positive qualities. Under conditions of high perceived stress, the moderating role of mindfulness in the mediation pathway between the teacher-student relationship and grateful disposition may fail; under low perceived stress, mindfulness might positively influence the relationship between the teacher-student relationship and social and emotional skills.

### 4.3 Limitations

This study, grounded in moral consensus theory and the significant others theory, explored the influence mechanism of teacher-student relationships on the moral consensus of primary school students. Unlike previous research, this study is the first to investigate the relationship between teacher-student relationships and moral consensus, incorporating social and emotional skills, grateful disposition, and mindfulness into a single model for examination. The findings enhance the understanding of the mechanism through which teacher-student relationships influence moral consensus and provide practical suggestions for enhancing moral consensus among primary school students.

Although this study possesses certain theoretical and practical significance, it still has shortcomings and limitations. Firstly, as a cross-sectional study, it did not employ a longitudinal follow-up design; therefore, causal inferences should be drawn with caution. Secondly, the study did not include other important variables (such as parent-child relationships) or explore the interactive effects of relationships with different significant others. Finally, the research data were collected solely from Qiannan Prefecture and Qiandongnan Prefecture in Guizhou Province, limiting the sample's representativeness. Future research could attempt to incorporate more potential influencing factors, such as primary school students' internet usage time and content, adopt longitudinal designs, expand the sample scope, and improve the study's generalizability and precision to provide theoretical and practical support for children's moral development.

## 5 Conclusions

In summary, the present study found that: (1) Teacher-student relationships had a significant positive predictive effect on the moral consensus of primary school students; (2) Both social and emotional skills and grateful disposition played mediating roles in the relationship between teacher-student relationships and the moral consensus of primary school students; (3) The indirect effect of teacher-student relationships on primary school students' moral consensus through social and emotional skills was moderated by mindfulness, whereas the indirect effect through grateful disposition was not moderated by mindfulness.

## Data Availability

The original contributions presented in the study are included in the article/supplementary material, further inquiries can be directed to the corresponding author/s.
